# Environmental heterogeneity, dispersal mode, and co-occurrence in stream macroinvertebrates

**DOI:** 10.1002/ece3.470

**Published:** 2013-01-09

**Authors:** Jani Heino

**Affiliations:** 1 Finnish Environment Institute, Natural Environment Centre, Ecosystem Change UnitP.O. Box 413, FI-90014, Oulu, Finland; 2 Department of Biology, University of OuluP.O. Box 3000, FI-90014, Oulu, Finland

**Keywords:** Co-occurrence, dispersal, environmental heterogeneity, headwater streams, metacommunities

## Abstract

Both environmental heterogeneity and mode of dispersal may affect species co-occurrence in metacommunities. Aquatic invertebrates were sampled in 20–30 streams in each of three drainage basins, differing considerably in environmental heterogeneity. Each drainage basin was further divided into two equally sized sets of sites, again differing profoundly in environmental heterogeneity. Benthic invertebrate data were divided into three groups of taxa based on overland dispersal modes: passive dispersers with aquatic adults, passive dispersers with terrestrial winged adults, and active dispersers with terrestrial winged adults. The co-occurrence of taxa in each dispersal mode group, drainage basin, and heterogeneity site subset was measured using the C-score and its standardized effect size. The probability of finding high levels of species segregation tended to increase with environmental heterogeneity across the drainage basins. These patterns were, however, contingent on both dispersal mode and drainage basin. It thus appears that environmental heterogeneity and dispersal mode interact in affecting co-occurrence in metacommunities, with passive dispersers with aquatic adults showing random patterns irrespective of environmental heterogeneity, and active dispersers with terrestrial winged adults showing increasing segregation with increasing environmental heterogeneity.

## Introduction

Understanding patterns and underlying mechanisms of species co-occurrence is a core area of community ecology. In general, biotic (e.g., interspecific competition), abiotic (e.g., environmental heterogeneity), and historical (e.g., differential colonization) factors are considered important for the co-occurrence of species among sites (Diamond 1975; Gilpin and Diamond 1982; Belyea and Lancaster 1999; Gotelli and McCabe 2002). Considering interspecific competition, Diamond (1975) coined a number of “assembly rules”, revolving around the idea that competing species should not occur together. This idea has subsequently been tested in various organisms groups, environmental contexts and geographical regions, and a meta-analysis of a large number of data sets revealed that species tend to coexist less than expected by chance (Gotelli and McCabe 2002). Although interspecific competition is often invoked as the mechanism leading to segregated patterns of species distributions, it is only one factor that may cause species to coexist less than expected by chance (Gilpin and Diamond 1982; Belyea and Lancaster 1999; Ulrich 2004).

A second reason for the degree of co-occurrence of species is related to environmental heterogeneity across a set of sites (Bell 2001; Reitalu et al. 2008). Given that different species typically have different niches (Hutchinson 1957; Chase and Leibold 2003), they should have differing optima along environmental gradients (i.e., niche position) and tolerance of environmental conditions (i.e., niche breadth). Hence, any two species differing in environmental responses are expected to be, at least partially, segregated across a set of sites if there is variation in environmental conditions (Bradley and Bradley 1985; Peres-Neto 2004). If segregated distributions are indeed due to differences in the environmental responses of species, increasing environmental heterogeneity among sites should lead to less co-occurrence. This idea can easily be tested using data sets from regions differing in environmental heterogeneity or subsets of sites within a region differing in environmental heterogeneity. However, few direct tests of the idea have been conducted, and the importance of environmental heterogeneity among sites for species co-occurrence has typically been indirectly inferred (McCreadie et al. 1997; Heino 2005a; Reitalu et al. 2008). However, a recent study in stream systems showed that within-drainage basin environmental heterogeneity affected the degree of taxon co-occurrence, with random distributions emerging across a set of sites with low environmental heterogeneity and significantly segregated distributions emerging across a set of sites with high environmental heterogeneity (McCreadie and Bedwell 2012).

A third potential reason to account for the degree of co-occurrence of species relates to differential colonization and extinction history (Gilpin and Diamond 1982; Jenkins, 2006). Although testing the effects of colonization and extinction directly requires monitoring a set of sites during several years and is highly problematic for multispecies assemblages, potential colonization ability and/or dispersal mode could be used as a proxy in this context (e.g., Jacobson and Peres-Neto 2010). One could envisage that passive dispersers are less segregated than active dispersers, because the distributions of the former are more random than those of the latter due to slow recolonization of denuded sites. By contrast, active dispersers may show active habitat selection, being able to recolonize denuded sites and occur more frequently at environmentally optimal sites.

Environmental heterogeneity and dispersal mode may also interact in affecting species co-occurrence. Because active dispersers with terrestrial adults should be able to track environmental variation better than passive dispersers with aquatic adults (Thompson and Townsend 2006; Heino 2011), they should show more segregation if (i) environmental heterogeneity among sites is high and (ii) the environmental responses of species are different. Passive dispersers with aquatic adults should show more random co-occurrence patterns, because they cannot effectively track environmental variation and may thus be absent in some suitable sites. This reasoning may also apply to groups of species comprising passive dispersers with terrestrial adults. To my knowledge, no one has tested the interactions between environmental heterogeneity and dispersal mode as possible determinants of species co-occurrence, although it is straightforward to sample sets of sites in different environmental contexts, measure environmental factors in the field and quantify environmental variation using modern multivariate methods (for suitable analytical methods, see Anderson et al. 2011). However, it is typically much more difficult to measure dispersal directly for many species in a metacommunity, and proxies for dispersal are almost inevitable (e.g., Jacobson and Peres-Neto 2010).

Small drainage basins provide amenable model systems for testing the above conjecture. They provide (i) a meaningfully defined abiotic template (i.e., streams in the same climatic regime) and (ii) biotic context (i.e., a regional species pool) for ecological studies (e.g., Peres-Neto 2004), (iii) streams within a drainage basin often differ sharply in environmental conditions (e.g., Heino 2005a), (iv) a drainage basin can be considered to support a metacommunity of interacting species (e.g., Brown and Swan 2010) and (v) they typically support macroinvertebrate assemblages with species varying widely in important traits (e.g., Tachet et al. 2000). Previous studies on the assembly rules of stream communities have typically assumed interspecific competition to be the underlying cause of the segregation of species (e.g., Heino 2005a). However, other stream studies have challenged the competitive explanation underlying species co-occurrence, and argued that the degree of co-occurrence of species is more likely to be related to the individualistic environmental responses of species (McCreadie et al. 1997; Peres-Neto 2004; Heino 2009; McCreadie and Bedwell 2012). The aim of the present study is to extend on the previous findings of the co-occurrence of species, and to examine the effects of changing environmental heterogeneity and dispersal mode on the co-occurrence of species. Thus, the aim is not to claim that interspecific competition (e.g., Gotelli and McCabe 2002) or predation (e.g., Englund et al. 2009) are not important for the segregation of species, but that environmental heterogeneity and/or mode of dispersal may also mediate the strength of these biotic interactions and lead to segregated distributions.

Specifically, I asked the following questions: (i) Is environmental heterogeneity related to the degree of species co-occurrence in drainage basins differing profoundly in environmental variation and within subsets of sites in each drainage basin? (ii) Are there differences in the level of species co-occurrence among groups of species differing in mode of dispersal? (iii) Do environmental heterogeneity and mode of dispersal interact in affecting species co-occurrence? I hypothesized that increasing environmental heterogeneity among sites should lead to increased species segregation due to the fact that more niche opportunities are available for individual species. I also expected that active dispersal should lead to increased segregation of species, because active dispersers are able to track environmental variation better than passive dispersers. I tested these expectations using data on stream macroinvertebrates from three high-latitude drainage basins.

## Materials and methods

### Characteristics of the study systems

The three study drainage basins, Iijoki (centered on 65^o^N, 27^o^E), Koutajoki (66^o^N, 29^o^E) and Tenojoki (70^o^N, 27^o^E), are in northern Finland. The sets of streams in each study drainage basin are characterized by extremely high environmental heterogeneity among sites, although overall among-drainage basin differences are also evident in this respect (S. Schmera, T. Eros, J. Heino, unpublished manuscript). The drainage basins studied support poor to moderately diverse stream macroinvertebrate communities in terms of species richness, beta diversity, and trait diversity, with the Tenojoki drainage basin being the least diverse and the Koutajoki drainage basin the most diverse (S. Schmera, T. Eros, J. Heino, unpubl. ms.). The regional species pools and individual streams are characterized by very low species-to-genus and species-to-family ratios, implying that only a few congeneric species are present and potentially coexist at a stream site (Heino 2005b). Furthermore, the macroinvertebrate communities are strongly dominated by insects, with more than 90% of species being insects in the headwater streams of each drainage basin. Previous research on the community-environment relationships of stream macroinvertebrates has implied that spatial patterns vary among high-latitude drainage basins (Heino et al. 2012), suggesting that patterns of species co-occurrence should also vary between the drainage basins.

The southernmost study area was located in the Iijoki drainage basin. This drainage basin is characterized by middle boreal coniferous forest and peatlands. Headwater streams in the drainage basin are often modified by forestry, drainage, and log-floating, although some near-pristine running waters are also present and were, in fact, selected as sampling sites. Near-pristine sites were not affected by drainage or log-floating, although minor forestry activities are present in their catchments. Twenty first-to-third order streams in the Iijoki drainage basin were surveyed in late May 2009. The total area of the Iijoki drainage basin is 14191 km^2^ of which the present study area covered about 2150 km^2^.

The easternmost study area is located in the Koutajoki drainage basin. Vegetation ranges from northern boreal coniferous forests to mixed-deciduous riparian woodlands, and from nutrient-poor bogs to fertile fens. These factors also provide the basis for a high variability in stream habitats across the drainage basin. Headwaters in the drainage basin are generally near-pristine. Twenty, first-to-third order streams were surveyed in the Finnish part of the Koutajoki drainage basin in late May 2008. The total area of the Koutajoki drainage basin is ca. 24500 km^2^ of which the present study area covered about 150 km^2^.

The northernmost study area was located in the Tenojoki drainage basin. This subarctic study area is characterized by arctic-alpine vegetation, comprising mountain birch (*Betula pubescens* ssp. *czerepanovii*) woodlands at low altitude and barren fell tundra at higher altitude. Headwater streams in the drainage basin range from near-pristine to pristine, as forestry and associated land uses are not generally feasible at these latitudes. Thirty, first-to-fourth order streams were surveyed in the Tenojoki drainage basin in the first half of June 2010. The total area of the Tenojoki drainage basin is 16386 km^2^ of which this study area covered about 5370 km^2^.

### Measurement of environmental variables

Several riparian, in-stream and water chemistry variables were measured at each site (see Heino et al. 2012). Each study site comprised an area of about 100 m^2^. Percentage cover of deciduous trees was assessed in a 50-m section on both banks directly upstream of the sampling site. Shading was estimated visually as percent canopy cover at 20 locations along transects (the number of which depended on stream width) at the whole study section. Current velocity (at 0.6 × depth) and depth were measured at 30 random locations along cross-stream transects, the number of which depended on stream width. Stream wetted width was measured at each site based on five cross-stream transects. Moss cover (%) and substratum particle class cover (%) were assessed in 10 randomly spaced 50 × 50 cm quadrats. Visual estimates of the percentage cover of five particle size classes were made for each quadrat using a modified Wentforth scale: (i) sand (diameter 0.25–2 mm), (ii) gravel (2–16 mm), (iii) pebble (16–64 mm), (iv) cobble (64–256 mm), (v) boulder (256–1024 mm). Mean values were used in the analyses. Standard deviations of velocity, depth, and moss cover were also used as indices of local, site-level habitat heterogeneity. Water samples were collected simultaneously with the field sampling, and they were analyzed for pH, conductivity, water color, and total phosphorus using Finnish national standards (National Board of Waters and the Environment 1981). Water color and total phosphorus were not measured in the Tenojoki basin due to logistical problems and negligible variation among sites. Most environmental variables showed considerable variation among the surveyed sites (Table 1), and all environmental variables (except water color and total phosphorus) were used in the multivariate analyses described below.

**Table 1:** Variation in the environmental variables within the three drainage basins. Variables are given as mean, min, max and standard deviation (SD)

	Iijoki (*n* = 20)				Koutajoki (*n* = 20)				Tenojoki (*n* = 30)			
	
	Mean	Min	Max	SD	Mean	Min	Max	SD	Mean	Min	Max	SD
Deciduous trees (%)	35	5	80	19	44	10	75	15	99.9	98	100	0.4
Shading (%)	34	10	70	20	44	5	85	26	16	0	55	14
Velocity (m/s)	0.40	0.16	0.73	0.16	0.51	0.21	1.02	0.19	0.37	0.14	0.58	0.11
Depth (cm)	24	16	35	7	25	10	46	10	19	13	33	19
Width (cm)	304	100	650	131	299	78	1200	266	574	88	2400	506
Macrophytes (%)	44	1	78	23	11	0	43	15	4	0	16	4
Sand (%)	10	0	49	12	11	0	73	18	1	0	22	1
Gravel (%)	6	0	37	9	9	0	30	8	2	0	25	5
Pebble (%)	10	0	55	14	33	0	64	19	15	1	65	12
Cobble (%)	29	2	53	14	26	0	61	16	45	10	81	20
Boulder (%)	44	0	82	25	20	0	92	24	37	1	83	22
pH	6.4	5.7	6.9	0.3	7.3	6.8	7.9	0.3	6.6	6.3	6.7	0.1
Conductivity (mS/m)	2.09	1.50	3.10	0.41	6.99	2.75	17.50	3.75	1.83	1.20	2.40	0.32

### Macroinvertebrate sampling, processing and identification

Stream macroinvertebrates were sampled between late May and mid June, depending on the latitude of a drainage basin. This is the season when the majority of macroinvertebrates in northern streams are still in the larval stage; however, they are already large enough to be identified to the lowest feasible level.

At each site, the field crew took a 2-min kick-net (net mesh size 0.3 mm) sample covering most microhabitats present in a riffle of approximately 100 m^2^. Microhabitats were visually assessed based on variation in velocity, depth, moss cover and particle size, and four 30-second and one-meter subsamples were divided among the main microhabitats in a riffle site. The subsamples were pooled in the field to provide the 2-min collective sample for each site. This sampling effort typically yields more than 70% of species occurring at a headwater site in a given season, mainly missing species that are only occasional in streams (Mykrä et al. 2006). Macroinvertebrates and associated material were immediately preserved in 70% alcohol in the field, and the samples were taken to the laboratory for further processing and identification. Macroinvertebrates were mainly identified to species (67.1% of taxa were species), but species group (11.3% of taxa were species group) and genus (21.6% of taxa were genera) levels were also used. Species-level identification is not yet possible in the study region for some genera, comprising mostly nonbiting midges (Diptera: Chironomidae).

### Dispersal mode categorisation

Macroinvertebrates were assigned into one of three groups based on their overland dispersal mode (see also Bilton et al. 2001; Bohonak and Jenkins 2003; Van de Meutter et al. 2007). Species in the first group (AqPa) had aquatic adults and show passive overland dispersal (i.e., Turbellaria, Nematoda, Oligochaeta, Hirudinea, Gastropoda, Bivalvia, Aranea, Crustacea), the second group (TePa) had terrestrial winged adults with mainly passive dispersal mode (i.e., Diptera with small body size [Ceratopogonidae, Chironomidae, Simuliidae, Psychodidae, Dixidae, Culicidae]), and the third group (TeAc) had terrestrial winged adults with mainly active dispersal mode (i.e., Ephemeroptera, Odonata, Plecoptera, Megaloptera, Trichoptera, Coleoptera, Diptera with large body size [Tipuloidea, Empididae, Muscidae]).

Although there is likely to be much among-species variation within each dispersal mode group, these groups should, on average, differ in their ability to actively locate environmentally suitable streams. For example, members of AqPa cannot actively disperse over land and thus cannot actively locate environmentally suitable streams. Although some water mites may travel on the adults of aquatic insects for considerable distances (Smith and Oliver 1986; Bohonak et al. 2004), dispersal is not active from the view of a mite, but directed by an adult insect. Being generally small in size, TePa species are mostly distributed by strong winds, thus comprising a strong random component in their distributions. Although some blackflies, for example, are known to disperse considerable distances, long-distance dispersal is not strictly active, but mediated by wind (e.g., Crosskey 1990). By contrast, being larger in size than species in the previous group, TeAc species are able to fly actively some distances and thus have better potential to locate environmentally suitable streams in a drainage basin. In summary, these dispersal mode groups should be related to the degree of randomness in the distribution of species in relation to the dispersal process. Randomness should decrease in the following order: AqPA > TePa > TeAc.

### Statistical methods for treating environmental data

The environmental data were first analyzed using a set of three multivariate methods to examine variation in environmental conditions among and within-drainage basins. Euclidean distance matrices were calculated from data on standardized environmental variables prior to the analyses below. (1) Principal coordinates analysis (PCoA; Gower 1966) was then used to visualize the scatter of sites based on their environmental conditions in the unconstrained ordination space. PCoA places the sites onto Euclidean distance ordination axes using a matrix of inter-point dissimilarities (Anderson et al. 2008). (2) Canonical analysis of principal coordinates (CAP; Anderson and Robinson 2003) was used to test for average differences in environmental conditions among the drainage basins. CAP aims to find axes through the multivariate cloud of points that are best in discriminating among a priori groups. CAP supplements the information provided by PCoA in that it helps one to discover among-group differences in constrained ordination space (“drainage basin” was used as a constraining factor). The null hypothesis of no differences between group centroids was tested using a permutation test with 999 runs. (3) Tests of homogeneity of dispersion (PERMDISP; Anderson 2006) were used to examine the multivariate dispersions in stream environmental conditions within each drainage basin (i.e., environmental heterogeneity across a set of sites). PERMDISP further uses the ANOVA F-statistic to compare among-group differences in the distance from observations to their group centroid. Significance of among-group differences is tested through permutation of least-squares residuals. The null hypothesis that there are no differences in within-drainage basin environmental variation among-drainage basins was tested using a permutation test with 999 runs.

Second, each within-drainage basin data were divided into two groups using distance of each site to the drainage basin centroid from the previous PERMDISP analysis (see above). Thus, in each drainage basin, the low heterogeneity site subsets included half of the sites with short distances to drainage basin centroid, and the high heterogeneity site subsets comprised the other half of the sites with higher distances to drainage basin centroid. The high and low heterogeneity groups were, in a way, arbitrary, as half of the sites with higher distance to a drainage basin centroid were designated to comprise the high heterogeneity site group, and the other half of the sites with shorter distance to a drainage basin centroid were designated to comprise the low heterogeneity group. Thus, there was no clear boundary value of distance to centroid in this regard, but the data were only divided into two equally sized parts in each drainage basin. PCoA was again used to visualize the distribution of sites in unconstrained ordination space, and PERMDISP was used to test quantitatively for differences in environmental heterogeneity between the low hetero-geneity and high heterogeneity site subsets within each drainage basin. Low heterogeneity site subset should thus include much less environmental variation than the high heterogeneity site subset across the streams. This can be visualized even in two-dimensional ordination plots, where most sites in the low heterogeneity subset are likely to be located within the high heterogeneity subset. However, this may not always be apparent in two-dimensional ordination solutions. PCoA, CAP, and PERMDISP were run using PERMANOVA+ for PRIMER (Anderson et al. 2008).

### Co-occurrence analysis

All co-occurrence analyses were based on presence-absence data, where rows were species and columns were streams. The C-score (Stone and Roberts 1990) was used to describe patterns of co-occurrence in each drainage basin, environmental heterogeneity subset within each drainage basin, and for each dispersal mode group. The C-score measures the degree of average pairwise co-occurrence, and if species are segregated according to environmental conditions in the present case, then the C-score should be larger than expected by chance. That is, the larger the C-score, the less the average pairwise co-occurrence of species. The number of checkerboard units (CU) for each species pair is:


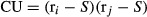


where *S* is the number of sites containing both species, and r_*i*_ and r_*j*_ are the matrix row totals for species *i* and *j*. The C-score is then averaged across all possible checkerboard pairs, and it is calculated for species that occur at least once in the matrix (Stone and Roberts 1990). Of the available measures of species co-occurrence, the C-score has been shown to have the greatest statistical power for detecting nonrandomness (Gotelli 2000).

The significance of the C-score was tested using the fixed-fixed null model, where the row and column sums were fixed. In such a null model, each random stream site contained the same number of species as the original stream site and each species occurred in the same frequency as in the original assemblage (Gotelli and Ellison 2002). No column weights were used in the analyses, as the potential effects of environmental heterogeneity on the co-occurrence of species were included in the study design (i.e., the three drainage basins and subset of sites in each drainage basin differing in environmental heterogeneity).

For the null model, random matrices were produced by shuffling the original matrix through repeated swapping of random submatrices (Manly 1995). This algorithm has good statistical properties, especially when used with the C-score, and it has a low propensity for Type I and Type II errors (Gotelli 2000). In analyses of co-occurrence for each drainage basin, site subset, and dispersal mode group combination, 5000 random matrices were constructed and mean and standard deviation for the index values thus obtained were calculated. Furthermore, a series of 30,000 “burn-ins” of initial swaps of transient effects were conducted prior to the construction of the 5000 random matrices. This number of swaps is considered adequate for small-to-moderate-sized data sets (Gotelli and Ulrich 2011), although very large data sets may require as much as 50,000 swaps (Fayle and Manica 2010). Statistical significance was then assessed by comparing the observed index value from the original matrix to the distribution of values derived from the random matrices (Manly 1995). Finally, to facilitate comparison between the different combinations of data and other studies, a standardized effect size (SES) (Gurevitch et al. 1992) was calculated as (Gotelli and McCabe 2002):(observed C-score–mean simulated C-score)/standard deviation of simulated C-scores, which indicates the number of standard deviations that the observed C-score is above or below the mean C-score from simulated matrices. SES values were used as the basis of ecological interpretation, as pure values of C-scores are affected by matrix size. High SES of the C-score means less co-occurrence than low SES values. Species co-occurrence analyses and associated randomization tests were conducted using EcoSim7 (Gotelli and Entsminger 2006).

All species in the total species matrix of each dispersal mode group in each drainage basin were used in the analyses of the low and high heterogeneity subsets of sites. This decision was made according to personal experience with the study system, because some species occurring in the total site set for a drainage basin may be temporarily absent in one or more sites in the subset site sets. Thus, the input matrices for the subsets of sites included some “missing species”, and such “degenerate” matrices thus included some empty rows. Thus, co-occurrence analyses started with a species list expected for subsets of sites within each drainage basin, as missing species may be important in the randomizations of null model analyses (Gotelli and Entsminger 2006). In a metacommunity context, this reasoning makes sense, as species may be temporarily absent in some sites; however, they recolonize them again afterwards.

## Results

### Patterns of environmental heterogeneity among-drainage basins and within each drainage basin

**Figure 1:**
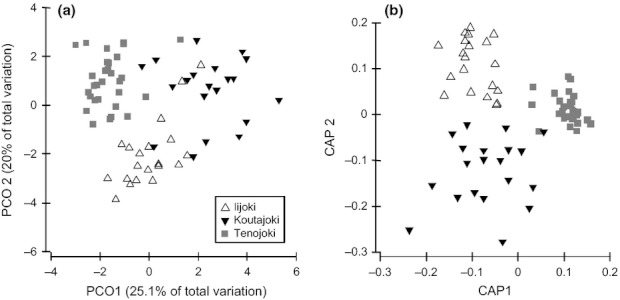
Scatter of sites in the environmental (a) PCoA and (b) CAP ordination plots across the drainage basins. The sites belonging to different drainage basins are shown by different symbols.

There was much variation in environmental variables among and within the drainage basins (Table 1), and this variation was also seen in the scatter of sites in the two-dimensional environmental PCoA and CAP ordinations (Fig. 1). Despite such variation, the three drainage basins differed in average overall environmental conditions based on CAP (trace = 1.534, *P* = 0.001). Furthermore, the drainage basins differed in within-drainage basin environmental heterogeneity based on PERMDISP (*F* = 8.568, *P* = 0.001), with the Koutajoki drainage basin showing highest environmental variation (mean Euclidean distance to group centroid ± SE: 3.874 ± 0.320), followed by the Iijoki drainage basin (3.017 ± 0.220) and the Tenojoki drainage basin (2.551 ± 0.169).

**Figure 2:**
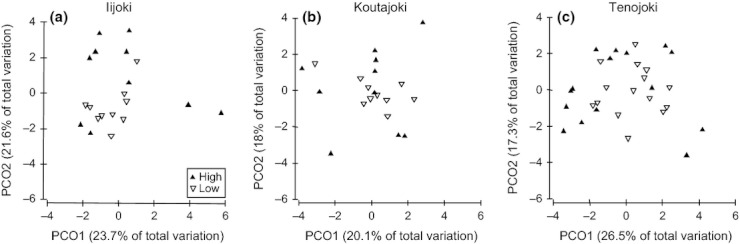
Scatter of sites in environmental PCoA ordination plot in each drainage basin. The sites belonging to the low and high heterogeneity site subsets are shown by different symbols. High = high environmental heterogeneity site subset, Low = low environmental heterogeneity site subset.

Within each drainage basin, PCoA (Fig. 2) showed that the sites in the high heterogeneity site subsets showed much more variation than those in the low heterogeneity site subset, and this was evident even in the two-dimensional ordination plots. PERMDISP quantified these visual inspections. In the Iijoki drainage basin, the mean Euclidean distance to group centroid was clearly higher for the high heterogeneity subset than the low heterogeneity subset (*F* = 13.284, *P* = 0.003), and the same self-evident pattern was found for the Koutajoki drainage basin (*F* = 45.055, *P* = 0.001) and the Tenojoki drainage basin (*F* = 10.057, *P* = 0.007).

### Co-occurrence analyses

**Figure 3:**
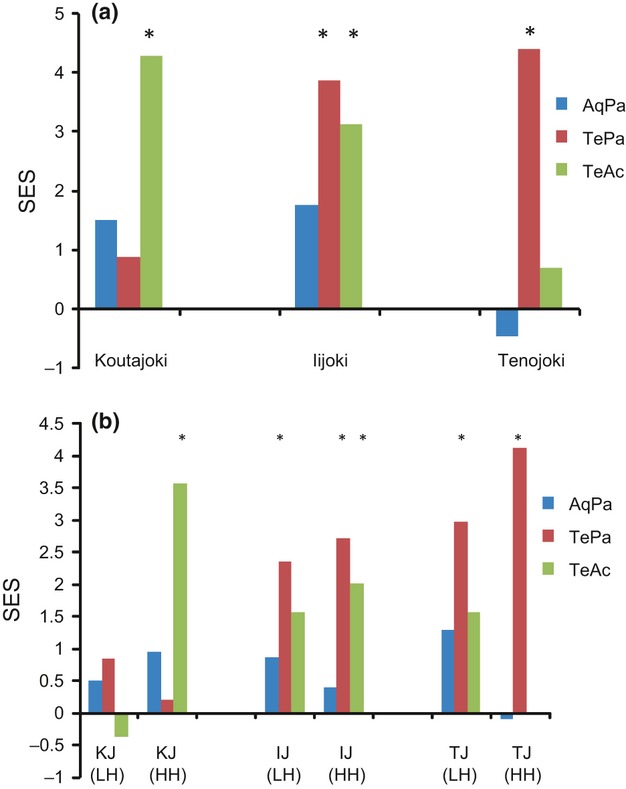
Variation in the standardized effect size (SES) values among the dispersal mode groups in different drainage basins (a) and among the dispersal mode groups in different environmental heterogeneity classes in each drainage basin (b). Dispersal mode groups: AqPa = Aquatic passive, TePa = terrestrial passive, TeAc = terrestrial active. Drainage basins: KJ = Koutajoki, IJ = Iijoki, TJ = Tenojoki. Habitat heterogeneity classes: LH = low heterogeneity, HH = high heterogeneity. Significant effect sizes are indicated by an asterisk.

In the across-all-sites analyses in each drainage basin (Table 2a, Fig. 3a), AqPa showed always random distributions. TePa showed a random distribution in the Koutajoki drainage basin, but showed significantly segregated distributions in the other two drainage basins. TeAc exhibited a random distribution in the Tenojoki drainage basin, but significantly segregated distributions in the other two drainage basins. Standardized effect size (SES) values were higher for TePa than TeAc in Iijoki and Tenojoki, but the opposite pattern was found in Koutajoki. SES values did not seem to vary according to the drainage basin, i.e., they did not generally follow among-drainage basin differences in environmental heterogeneity. However, TeAc showed “a structured pattern”, where SES varied along the environmental heterogeneity gradient across the drainage basins (Koutajoki > Iijoki > Tenojoki). The analysis with taxa identified to the species level only generally showed similar results, with AqPa showing random distributions, TePa showing random distributions in the Koutajoki basin and significantly segregated distributions in the other two basins, and TeAc showing random distributions in the Tenojoki basin and significantly segregated distributions in the other two basins (Appendix S1).

**Table 2:** Results of co-occurrence analyses. The analyses were based on the C-Score (Stone and Roberts 1990). Separate analyses were carried out for each dispersal mode group (a) across the whole set of sites in each drainage basin and (b) for the low and high environmental heterogeneity site subsets within each drainage basin (see text). The drainage basins are ordered from the most environmentally heterogeneous (Koutajoki) to the least environmentally heterogeneous (Tenojoki). Significant results are in bold

	C-score	Mean sim index	*P* (obs ≥ exp)	SES	Species
(a)					
Koutajoki					
AqPa	3.908	3.613	0.085	1.495	16
TePa	5.192	5.144	0.205	0.863	75
TeAc	8.886	8.693	**<0.001**	**4.285**	68
Iijoki					
AqPa	8.780	8.452	0.056	1.743	14
TePa	6.119	5.941	**0.001**	**3.867**	64
TeAc	6.778	6.620	**0.002**	**3.126**	63
Tenojoki					
AqPa	5.047	5.339	0.478	−0.492	7
TePa	10.145	9.734	**<0.001**	**4.392**	52
TeAc	8.576	8.483	0.221	0.666	39
(b)					
Koutajoki (low heterogeneity)					
AqPa	1.890	1.810	0.231	0.500	11
TePa	2.419	2.387	0.205	0.840	52
TeAc	3.191	3.201	0.629	−0.377	54
Koutajoki (high heterogeneity)					
AqPa	1.606	1.507	0.172	0.947	13
TePa	2.057	2.050	0.392	0.199	64
TeAc	2.482	2.390	**<0.001**	**3.550**	66
Iijoki (low heterogeneity)					
AqPa	3.833	3.759	0.212	0.849	13
TePa	2.202	2.121	**0.029**	**2.352**	52
TeAc	2.427	2.384	0.079	1.551	51
Iijoki (high heterogeneity)					
AqPa	1.890	1.845	0.305	0.398	11
TePa	2.646	2.566	**0.008**	**2.701**	46
TeAc	2.388	2.327	**0.040**	**1.997**	49
Tenojoki (low heterogeneity)					
AqPa	0.800	0.737	0.376	1.287	5
TePa	4.522	4.307	**0.007**	**2.954**	36
TeAc	2.764	2.654	0.073	1.549	32
Tenojoki (high heterogeneity)					
AqPa	4.300	4.357	0.414	−0.111	5
TePa	3.760	3.541	**0.002**	**4.120**	43
TeAc	3.522	3.523	0.475	−0.024	32

Abbreviations: Mean sim index = Mean simulated C-score from 5000 random runs, *P* (obs ≥ exp) = Probability of the observed C-score larger than expected C-Score from random runs, SES = standardized effect size. Species = number of species with at least one occurrence in the data set.

In the analyses of environmental heterogeneity subsets within each drainage basin (Table 2b, Fig. 3b), AqPa showed random co-occurrence irrespective of environmental heterogeneity. TePa showed random distributions in both subsets of sites in the Koutajoki drainage basin, but significantly segregated distributions in the other two drainage basins. If significantly segregated, higher SES values were found in the high environmental heterogeneity subsets of sites within the Iijoki and Tenojoki drainage basins. TeAc showed random patterns in the low heterogeneity site subsets and significantly segregated distributions in the high heterogeneity subsets in the Iijoki and Koutajoki drainage basins. In the Tenojoki drainage basin, co-occurrence was random regardless of environmental heterogeneity.

## Discussion

### Co-occurrence in relation to environmental heterogeneity

Most previous studies on species co-occurrence have been based on the underlying expectation that segregated distributions are caused by competitive (e.g., Diamond 1975) or predatory interactions (e.g., Englund et al. 2009). However, other explanations not directly related to local biotic interactions may also be underlying nonrandom distribution patterns. These include environmental heterogeneity and colonization history, which should be particularly important at large spatial scales and within a metacommunity (Thompson and Townsend 2006; Heino and Mykrä 2008; Brown and Swan 2010). I examined three environment- and dispersal-related questions for species co-occurrence in this study: (i) Is environmental heterogeneity related to species co-occurrence? (ii) Is dispersal mode related to species co-occurrence? (iii) Are environmental heterogeneity and dispersal mode interacting in this context? Both among-drainage basins and within-drainage basin comparisons were made to unravel the degree of species co-occurrence in relation to environmental heterogeneity.

In the among-drainage basin comparisons, species segregation for terrestrial active dispersers (TeAc) was clearly highest in the drainage basin with the highest environmental heterogeneity, whereas for the other two dispersal mode groups, no such association was found. This finding suggests that, for the group with most potential for active dispersal and capacity to track environmental variation, increasing environmental heterogeneity may indeed beget higher levels of species segregation. It is also notable that aquatic passive dispersers (AqPa) showed always random distributions, suggesting that environmental heterogeneity does not affect segregation or aggregation in this group of taxa with aquatic adults and passive dispersal mode. This finding is in agreement with the idea that weak dispersers are not able to track efficiently environmental variation across a set of sites (Thompson and Townsend 2006; Hájek et al. 2011), whereas they may be driven more by random or stochastic, disturbance-related extinction events and slow recolonization of denuded sites. Terrestrial passive dispersers (TePa) were intermediate between the other two dispersal mode groups in that they showed significant segregation in two of the drainage basins and, quite unexpectedly, a random pattern in the most environmentally heterogeneous drainage basin. This finding suggests that there may also be a large random component in the co-occurrence of TePa. This may be because species in this group are also randomly distributed (e.g., by strong winds) and thus may not necessarily show high degrees of active habitat selection.

In the within-drainage basin analyses, no consistent patterns were found between the subsets of low and high environmental heterogeneity. AqPa always showed random distributions irrespective of environmental heterogeneity. By contrast, TePa showed significantly segregated distributions in two of the drainage basins, and segregation was higher in the high environmental subset of sites. TeAc similarly showed significant segregation in two drainage basins and, within the drainage basins, only in the high environmental heterogeneity subset. Thus, although there was considerable context dependency in the results with regard to the drainage basin and dispersal mode group, the probability of finding high levels of segregation as measured using SES tended to increase with environmental heterogeneity within the drainage basins. This finding lends tentative support for the importance of environmental heterogeneity in underlying segregated distribution patterns in groups of species with active dispersal mode. In a similar vein, McCreadie et al. (1997) found that blackflies showed random patterns across a set of sites with low environmental variation, suggesting that environmental heterogeneity is necessary for segregated distributions or that blackflies are perhaps randomly distributed by strong winds. Furthermore, McCreadie and Bedwell (2012) recently found that segregation of insect genera was stronger in a set of sites with high environmental heterogeneity than in a set of sites with low environmental heterogeneity. The present findings thus extend on these two studies by showing (i) that both environmental heterogeneity and dispersal mode may interact in affecting co-occurrence and (ii) that there is variation in these patterns among-drainage basins.

The finding that environmental heterogeneity may be important in setting the levels of co-occurrence suggests that species environmental niche differences are underlying the pattern. Because streams are highly heterogeneous ecosystems within a drainage basin (Heino and Mykrä 2008; Heino et al. 2012), species should have multiple niche opportunities and different species should utilize these increased opportunities. In fact, species in highly contrasting systems, such as boreal and tropical streams, are clearly adapted to utilize this high environmental heterogeneity, as different species may have highly different niches in terms of both position and breadth (Heino 2005b; Siqueira et al. 2009). What is more striking is that potentially competing species, such as species belonging to the same genus, may have highly different environmental niches (e.g., Heino 2005b). The degree to which a species' realized niche (sensu Hutchinson 1957) deviates from its fundamental niche (sensu Hutchinson 1957) is difficult to judge, because the former also reflects the effects of biotic interactions (which may also be difficult to determine in a field-based descriptive study). However, there is at best only circumstantial evidence that competition or predation leads to the exclusion of macroinvertebrate species from a whole riffle site (Grant and Mackay 1969; Heino 2009), although both interference and resource competition may be clearly visible at smaller scales (Hemphill and Cooper 1983; McAuliffe 1984; Hemphill 1988; Dudley et al. 1990) and closely related species may be temporally segregated at the same site (Hynes 1961; Ulfstrand 1967; Grant and Mackay 1969). Thus, it may well be that the fundamental and realized niches of a given stream macroinvertebrate species are close to each other, and that species distributions across streams are largely governed by abiotic environmental factors.

Combining patterns with underlying mechanisms is never easy and often even risky, and this is particularly true for large-scale patterns of multispecies communities. Thus, one has to infer mechanisms from patterns rather than experimentally verify the potential effects of biotic interactions and other factors on co-occurrence patterns. The null model approach provides a potentially powerful means to aid in deciding if any factor may structure the distributions of species (Gotelli and Graves 1996; Gotelli and McCabe 2002). As mentioned in the introduction, the aim of the present study was not to dismiss the importance of biotic interactions, but to test if environmental heterogeneity is responsible for increasing the segregation of species across a set of sites. It is unrealistic to test this idea experimentally, as imitating natural environmental variation in highly heterogeneous and biologically diverse systems, such as headwater streams, is practically impossible. Thus, one needs to apply null models in drainage basins differing in environmental heterogeneity and subsets of sites within each drainage basin to infer if environmental heterogeneity and/or dispersal mode are associated with increased segregation of species across sites. Despite that testing this question is relatively easy provided that one has surveyed a large number of sites in a number of drainage basins, I know of no studies that have addressed the same question using a similar multidrainage basin approach.

### Characteristics and extensions of the approach

Both among-drainage basins and within-drainage basin comparisons of species segregation along environmental heterogeneity gradients are important. The former comparison provides information about not only the effects of environmental heterogeneity, but also possible species pool effects on species co-occurrence. If the identities of species or the occupancy frequencies of the same species in different drainage basins vary (McCreadie and Adler 2006), one should expect different patterns of species co-occurrence among-drainage basins as well. By contrast, in the within-drainage basin comparisons, the species pool is basically the same (although partly different species may occur in the low and high heterogeneity site subsets) and metacommunity-level processes (e.g., dispersal distances, corridors and rates) should not vary appreciably. Hence, one can control for the species pool effects on species co-occurrence using subsets of sites within a drainage basin.

Given enough replicate drainage basins, one could also run an ANOVA test for the effects of dispersal mode group, drainage basin and site subset (drainage basin) factors on the degree of co-occurrence among species (e.g., measured as SES). Alternatively, if one is interested only in differences in environmental heterogeneity across a large number of drainage basins, one could associate SES with environmental heterogeneity (e.g., measured based on PERMDISP) using regression analysis. Unfortunately, in the present example, data set, the number of drainage basins was too low for direct statistical comparisons of the degree of co-occurrence in relation to environmental heterogeneity. Data sets that cover a larger number of regions, comprising both species lists and environmental variables at the level of sites, are likely to be already available for at least mammals, birds, fish and vascular plants, so other researchers should test the idea proposed in this study in other organism groups, environmental settings and regional contexts. Statistically testing variation in SES values among different factors is common in meta-analyses (Gurevitch et al. 1992; Gotelli and McCabe 2002), and a similar approach could be applied in analyses of among-region variation in species co-occurrence in an organism group (for a recent example, see Krasnov et al. 2011).

### Conclusions

To conclude, environmental heterogeneity was related to species co-occurrence in a metacommunity, with higher environmental heterogeneity tending to beget higher levels of species segregation. These patterns were, however, contingent on both dispersal mode (e.g., AqPa showed always random distributions, TeAc were segregated mainly in the high heterogeneity contexts) and drainage basin (e.g., different dispersal mode groups were strongly segregated in different drainage basins). Such drainage basin-related context dependency is high in environmentally heterogeneous systems, such as high-latitude drainage basins. I thus urge other researchers to use a similar approach to study the effects of environmental heterogeneity on patterns in the co-occurrence of species, as such an approach is obviously important in advancing our understanding of community ecology.

## Supporting Information

**Appendix S1.** Results of co-occurrence analyses based on species-level data.
