# Supplementary material for: Environmental heterogeneity, dispersal mode, and co-occurrence in stream macroinvertebrates
Source: Ecol Evol. 2013 Jan 9;3(2):344–55. doi: 10.1002/ece3.470 (PMC3586644; doi:10.1002/ece3.470)
Supplement: Supplementary file 1 [file ece30003-0344-SD1.doc]

**Appendix 1. Results of co-occurrence analyses based on species-level data.**

Heino, J. Environmental heterogeneity, dispersal mode and co-occurrence in stream macroinvertebrates. Ecology and Evolution.

Macroinvertebrates were assigned into one of three groups based on their overland dispersal mode. In these additional analyses, only macroinvertebrates identified to the species level were included. Note that in these additional analyses blackflies (Diptera: Simuliidae) were moved to terrestrial active dispersal mode (TeAc). Species in the first group (AqPa) had aquatic adults and show passive overland dispersal (i.e., Oligochaeta, Hirudinea, Gastropoda, Bivalvia, Aranea, Crustacea), the second group (TePa) had terrestrial winged adults with mainly passive dispersal mode (i.e., Diptera: Chironomidae), and the third group (TeAc) had terrestrial winged adults with mainly active dispersal mode (i.e., Ephemeroptera, Odonata, Plecoptera, Megaloptera, Trichoptera, Coleoptera, Diptera: Simuliidae).

In each drainage basin , AqPa showed always random distributions, although the test was meaningless in the Tenojoki basin due to a small number of species with scattered occurrences. This was also the reason, why no within-basin analyses were run here. TePa showed a random distribution in the Koutajoki drainage basin, but was significantly segregated in the other two drainage basins. TeAc exhibited a random pattern in the Tenojoki drainage basin, but showed significantly segregated distributions in the other two drainage basins. Standardised effect size (SES) values were higher for TeAc than TePa in the Iijoki and Koutajoki basins, but the opposite was true in the Tenojoki basin. SES values varied relatively similarly to those of the original analyses (see Table 2).

Table A1: Results of additional co-occurrence analyses at the species level. Significant results are in bold.

|  | C-score | Mean sim index | P (obs ≥ exp) | SES | Species |
| --- | --- | --- | --- | --- | --- |
| **Koutajoki** |  |  |  |  |  |
| AqPa | 3.952 | 3.575 | 0.113 | 0.827 | 7 |
| TePa | 4.274 | 4.254 | 0.371 | 0.261 | 50 |
| TeAc | 8.925 | 8.707 | **<0.001** | **3.908** | 61 |
|  |  |  |  |  |  |
| **Iijoki** |  |  |  |  |  |
| AqPa | 10.857 | 10.397 | 0.071 | 1.632 | 7 |
| TePa | 5.971 | 5.732 | **0.020** | **2.371** | 38 |
| TeAc | 6.179 | 5.923 | **<0.001** | **4.657** | 61 |
|  |  |  |  |  |  |
| **Tenojoki** |  |  |  |  |  |
| AqPa | - | - | - | - | 4 |
| TePa | 7.558 | 7.149 | **0.002** | **3.372** | 40 |
| TeAc | 12.947 | 12.778 | 0.127 | 1.147 | 35 |

Abbreviations: Mean sim index = Mean simulated C-score from random runs, P (obs ≥ exp) = Probability of the observed C-score larger than expected C-Score from random runs, SES = standardised effect size. Species = number of species with at least one occurrence in the data set.
